# An *In-Vivo* Study of Effects of Platelet-Rich Plasma on Transforming Growth Factor-β1 and Matrix Metalloprotein 9 Expression in Traumatic Ulcers with Diabetes Mellitus

**DOI:** 10.1055/s-0043-1764429

**Published:** 2023-05-12

**Authors:** Desiana Radithia, Yuliana Yuliana, Yeni Puspitasari, Rossy Sismiyanti, Aulya Setyo Pratiwi

**Affiliations:** 1 Department of Oral Medicine, Faculty of Dental Medicine, Universitas Airlangga, Indonesia; 2 Oral Medicine Residency Program, Faculty of Dental Medicine, Universitas Airlangga, Indonesia

**Keywords:** diabetes mellitus, oral ulcer, platelet-rich plasma, TGF-β1, MMP-9

## Abstract

**Objective** Diabetes mellitus is not only characterized by alterations in the wound healing process but also during oral ulcer healing. The platelet-rich plasma (PRP) can be used to stimulate the healing process. This study was performed to analyze the effect of PRP on traumatic ulcers with diabetes mellitus in an animal model by analyzing the expression of transforming growth factor β1 (TGF-β1) and matrix metalloprotein 9 (MMP-9).

**Materials and Methods** The diabetes mellitus model was developed using streptozotocin that was administrated to *Rattus novergicus.* The traumatic ulcer model was obtained by placing a heated tip of a ball burnisher for 5 seconds on the lower mucosa labial. Then, the traumatic ulcer was treated with PRP for 3, 5, and 7 days. The expression of TGF-β1 and MMP-9 was analyzed with indirect immunohistochemistry, and differences between each marker were analyzed with statistical analysis.

**Results** All animals showed clinical oral ulceration as a yellow base during the experiment. The application of PRP showed a higher level of TGF-β1 expression than the controls for 3, 5, and 7 days ( *p* < 0.05). In contrast, the MMP-9 expression was lower than the control for 5 and 7 days ( *p* < 0.05).

**Conclusion** The PRP affected traumatic ulcers with diabetes mellitus by promoting healing through TGF-β1 expression and suppressing the MMP-9 expression. This material can serve to develop a promising topical therapy for traumatic ulcers, especially with an underlying disease such as diabetes mellitus.

## Introduction

Diabetes mellitus has a higher prevalence of oral mucosal disorders that has an association with chronic immunosuppression, 1 such as delayed wound healing and salivary hypofunction. 2 The most common mucosal wound is a traumatic ulcer; it is characterized by damage to the epithelial tissue and underlying connective tissue 3 due to mechanical injury, thermal, electrical or chemical burn. 4 The primary healing of traumatic ulcers is similar to wound healing in the skin, which causes delayed healing in patients with diabetes mellitus.

The topical therapy commonly used in traumatic ulcers is saline mouthwash, antiseptic mouthwash such as chlorhexidine gluconate 0.2%, nonsteroidal anti-inflammatory mouthwash such as benzydamine hydrochloride 0.15%, or steroids such as hydrocortisone sodium succinate in the form of a mucoadhesive buccal patch. 5 All these materials were unable to interfere with the healing pathways of traumatic ulcers, especially in diabetes mellitus. The other option as therapy is to use self-material-like platelet-rich plasma (PRP). This material is considered to stimulate the intrinsic pathway of healing. The PRP contains many growth factors, which are highly important for wound healing. These growth factors promote cell migration, proliferation, and differentiation, which are essential for wound healing. After activation in the wound area, platelets will release growth factors to regulate cell migration, proliferation, and matrix deposition and reduce the risk of infection from the wound, increasing the quality of healing and tissue repair. 6

During the healing process, the transforming growth factor β1 (TGF-β1) is the primary growth factor for re-epithelialization, angiogenesis, and fibroblast proliferation. In contrast, the matrix metalloproteinase 9 (MMP-9) is needed to balance the fibroblast proliferation and collagen deposition to prevent fibrosis. In diabetes mellitus patients, TGF-β1 and MMP-9 activity alteration occurs in delayed healing. 7

PRP preparations are available in the form of injections, 8 powder, 9 and gel. 10 Several studies showed that the PRP injection in non-healing oral ulcers achieved complete epithelialization after 8 weeks. 8 In the case of oral ulcers caused by oral pemphigus vulgaris, the healing process took 8 weeks to 24 months. 11 12 Due to a lack of information about the PRP's effects on traumatic ulcer healing, especially in diabetes mellitus, the current study analyzed the PRP influence on TGF-β1 and MMP-9 expression in traumatic ulcers and considered it a good material for traumatic ulcer therapy.

## Materials and Methods

### The Ethical Approval

The protocol of this study and the protocol for Animal Care and Use were approved by the Ethics Committee of health and research with registration number 776/HRECC.FODM/XII/2019.

### Experimental Materials

Thirty Wistar rats *(Rattus norvegicus)* were used that were randomly divided into six groups. Each group contained five animals. The animal adapted for 7 days with free access to food and drink. The rats were selected using criteria of 2 to 3 months, gender only male, the body weight of 150 to 250 grams, and health status (measured by the rat's liveliness, shiny hair, and glowing hair).

### PRP Preparation

The PRP was obtained from 35 Wistar rats by collecting blood after general anesthesia. Then, the blood was centrifuged two times at the speed of 4000 rpm. The plasma was collected after centrifugation.

The plasma was subjected to freeze-drying. Before this process, the plasma was frozen at −83°C for 24 hours. Then, PRP was lyophilized using a sublimation drying machine. The product was then mixed with 2% carboxymethylcellulose (CMC) in a ratio of 1:1 (w/w) to form a gel preparation.

### Diabetes Mellitus Model

The diabetes mellitus model was obtained by fasting the rats for 4 hours, and then administering streptozotocin (BioWorld with CAS number 18883-66-4, USA; 50 mg/kg body weight) diluted in a citric acid buffer (pH 4.5) intraperitoneally. After the administration of streptozotocin, all animals were given 10% dextrose peroral to avoid sudden hypoglycemia. 13 Lastly, the rats were observed for 3 days to develop diabetes mellitus; it was confirmed that fasting blood glucose resulted in ≥ 126 mg/dL glucose level.

### Traumatic Ulcer Model

After the animals were confirmed to have diabetes mellitus, the traumatic ulcer model was developed using the heated tip of a 3 mm ball burnisher. Before the procedure, the animals were anesthetized by intramuscularly injecting ketamine. 14 15

A 3-mm diameter ball burnisher was heated for ± 45 seconds. Then, the tip was gently placed in the lower oral mucosa as deep as the diameter of the burnisher tip for 1 second. The formation of a traumatic ulcer was confirmed if the ulcer showed damaged epithelium, the middle area showed yellowed whitish color, had a well-defined wound, irregular edge, and was surrounded by a red area. 14 15

### PRP Administration

After the traumatic ulcer developed, PRP gel was applied, while the control group was given 2% CMC only. After the treatment, the experimental animals were not given food and drink for 30 minutes so that the drug could be absorbed maximally. The PRP gel was applied once a daily for 3, 5 and 7 days. after the treatment, the animal was sacrificed by anasthesia using ketamine, and a biospy of lower labial mucosa of the animals was performed. Next, the tissue was immersed in a 10% formalin buffer solution with 10 times the tissue volume for 1 day. 16

### TGF-β1 and MMP-9 Expression

The expression of TGF-β1 and MMP-9 was studied by indirect immunohistochemistry. The monoclonal antibodies used were anti-TGF-β1 (TGF-β1 3C11, Santa Cruz Biotechnology Inc., USA) and anti-MMP-9 2C3 (MMP-9 2C3, Santa Cruz Biotechnology Inc., USA). The counterstaining was done using hematoxylin–eosin. A single oral pathologist studied each expression with a Nikon E100 microscope under 1000 magnification in five different fields of view.

### Statistics Analysis

The independent *t* -test was used to determine the difference in the expression of TGF-β1 and MMP-9 between the intervention and control groups on each day of observation. The significance value for all analyses was *p* < 0.05. Data were analyzed using Statistical Product and Service Solution (SPSS) version 24 (IBM SPSS Statistic 24 for Mac, New York, NY, USA).

## Results

### Traumatic Ulcer Observation

**Fig. 1:**
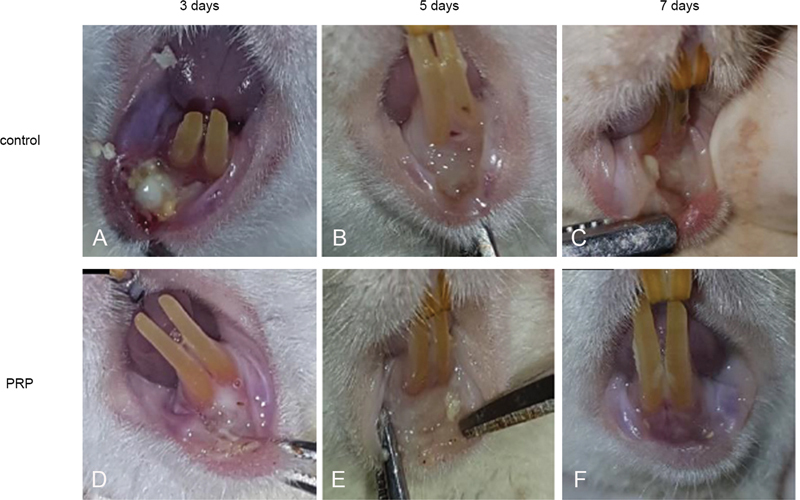
Clinical images of traumatic ulcers on the lower labial mucosa. ( **A** and **C** ) Three days; ( **B** and **D** ) 5 days and ( **C** and **E** ) 7 days.

The traumatic ulcer was observed in all groups at 3 days, 5 days, and 7 days. A yellow-colored traumatic ulcer appeared within 3 days of both control and PRF application ( Figs. 1A and 1D ). After 5 days, the traumatic ulcer partly healed with a reduced yellow area ( Figs. 1B and 1E ). After 7 days, the traumatic ulcer appeared to be completely healed, and the mucosa looked normal ( Fig. 1C and 1F ).

### TGF-β1 Expression

**Fig. 2:**
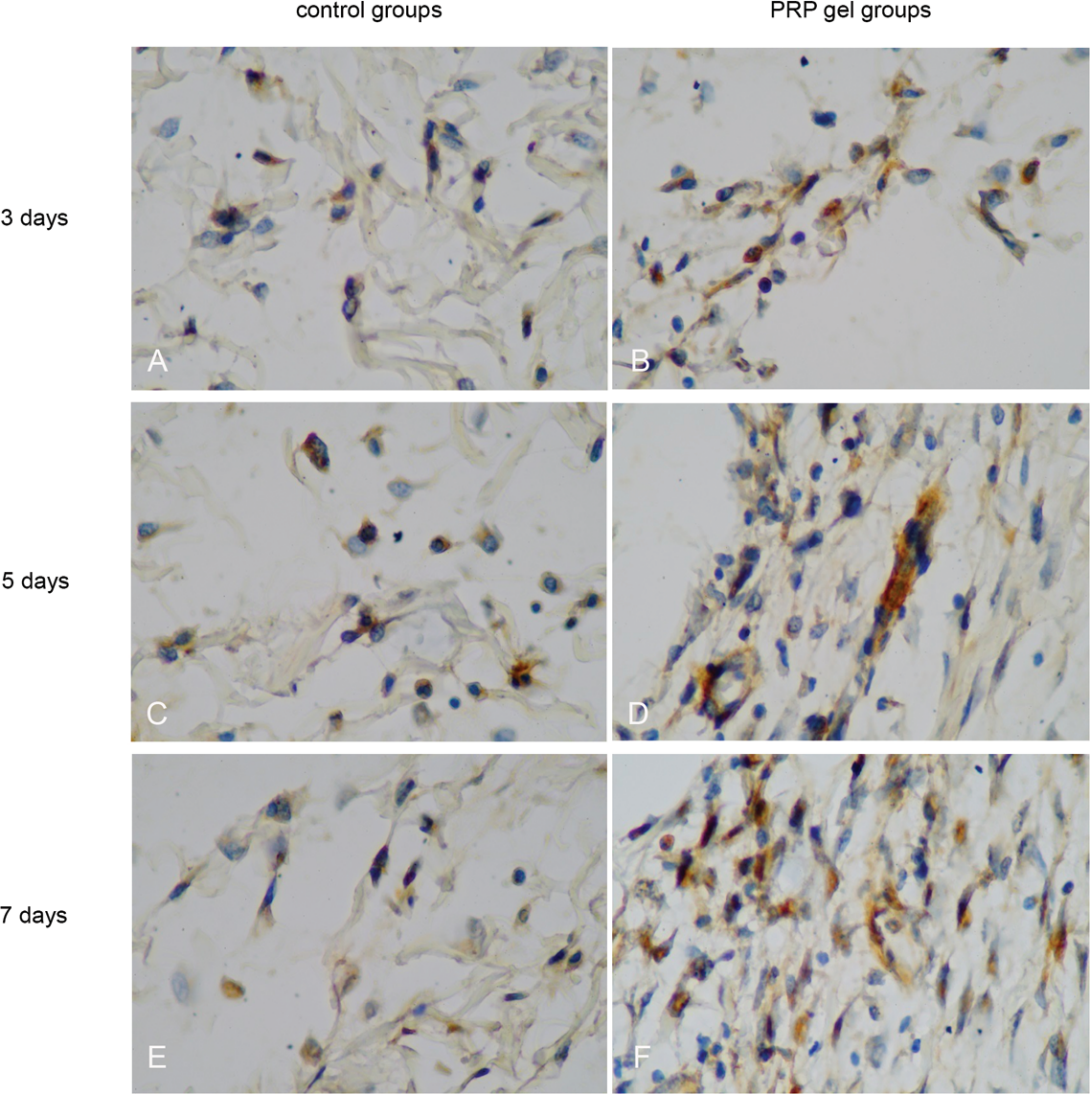
Microscopic of immunohistochemical examination results showing TGF-β1 with a light microscope with 1000x magnification.

**Fig. 3:**
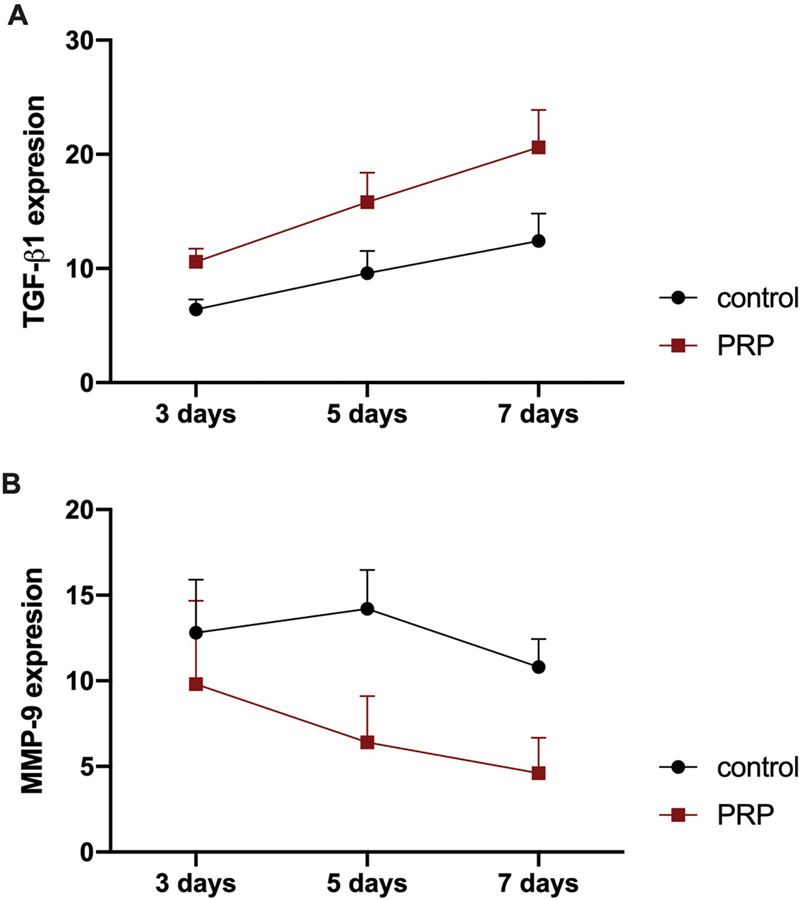
The expression marker in traumatic ulcer tissue from mucosa labial. ( **A** ) TGF-β1 ( **B** ) MMP-9 expression.

The TGF-β1 expression was observed in fibroblast cells ( Fig. 2 ). The application of PRF in TGF-β1 expression at 3 days, 5 days, and 7 days was higher compared with that in the control group ( *p* = 0.001; *p* = 0.003 and *p* = 0.002; respectively) ( Fig. 3A ).

### MMP-9 Expression

**Fig. 4:**
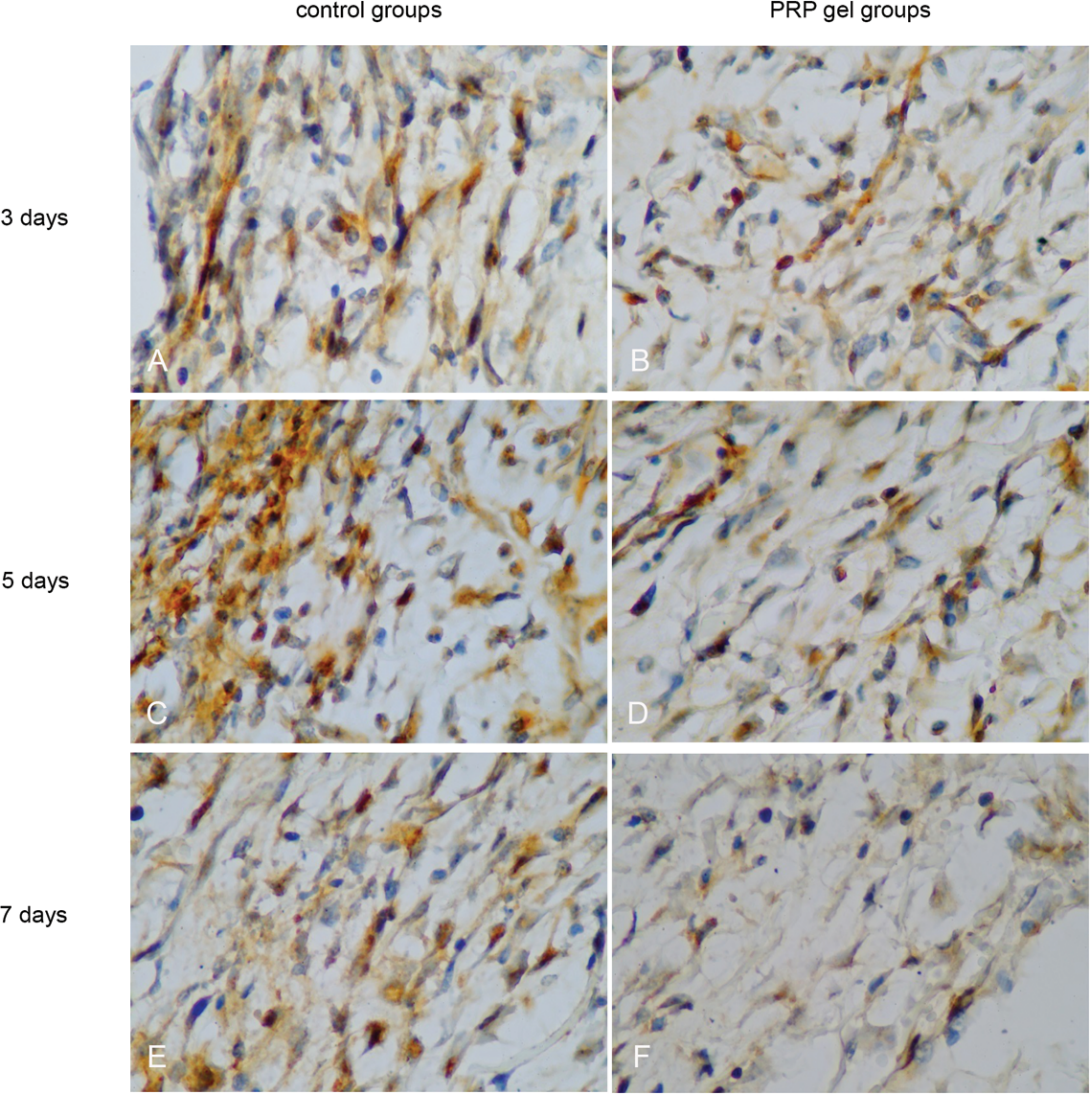
Microscopic immunohistochemical examination results show MMP-9 with a light microscope with 1000x magnification.

The MMP-9 expression was observed in fibroblast cells ( Fig. 4 ). The application of PRF in MMP-9 expression at 5 days and 7 days was lower compared with that in the control group ( *p* = 0.001; *p* = 0.001, respectively). While 3 days resulted in no difference in MMP-9 expression compared with the control group ( *p* = 0.285) ( Fig. 3B ).

## Discussion

Diabetes mellitus results in delayed healing due to the formation of increased advanced glycation end-products (AGEs) due to hyperglycemia. The binding process of Age to the AGE receptor (RAGE) increased reactive oxygen species (ROS). Excessively increasing ROS exceeds the antioxidant capacity, resulting in oxidative stress. Oxidative stress triggers the activation of nuclear factor kappa B (NF-kB), followed by an increase in the production of proinflammatory cytokines such as tumor necrosis factor α (TNF-α), interleukin 1β (IL-1β), interleukin 6 (IL-6), and MMP-9. All these cytokines shift the macrophage polarization to the M1 type. The imbalanced M1/M2 ratio imbalance results in a prolonged inflammatory phase so that the ulcer becomes chronic, and the healing process is delayed. 17

After the administration of topical PRP, improvement was noticed until 7 days when the ulcer healed maximally, as compared with the control group, which consisted of traumatic ulcers. Topical administration of PRP provides an environment of growth factors and cytokines that can stimulate and initiate the healing process, including platelet-derived growth factor (PDGF) from platelets, transforming growth factor-β (TGF-β), epithelial cell growth factor (ECGF), insulin-like growth factor (IGF), and vascular endothelial growth factor (VEGF). These growth factors will stimulate the macrophages to shift into the M2 type, thus causing the proliferation of fibroblasts and the synthesis of collagen. 11 The PRP contains 50 to 80% alpha granules protein (adhesive protein), one of which is TGF-β1. 18 This research showed an increased level of TGF-β1, which was in line with the study from Boakye *et al* . Besides containing TGF-β, PRP increased the expression of TGF-β. 19 PRP activates new platelets when it encounters tissue activators (calcium, collagen) 20 and releases various bioactive proteins that include growth factors, one of which is TGF-1. Platelets contain 95% of TGF-β1 from total platelets. Administration of PRP increases the amount of TGF-β1 in macrophage cells, which in turn transcribes NF-kB in the macrophage cell nucleus, decreasing the production of proinflammatory cytokines such as TNF-α, IL-1β, IL-6, and MMP-9. TGF-β1 in wound healing regulates not only epithelialization but also inflammation, angiogenesis, and granulation tissue formation. TGF-β1 is shown to be an important player in all phases of wound healing by regulating the functions of keratinocytes, fibroblasts, endothelial cells, monocytes, and other cell types. 21

TGF-β1 is an essential player in all phases of wound healing by regulating the functions of keratinocytes, fibroblasts, and endothelial cells. Although many growth factors also play a role in keratinocyte migration during wound healing, TGF-β1 has more roles than other growth factors. After the injury, TGF-β1 is rapidly regulated and released by keratinocytes, platelets, monocytes, macrophages, and fibroblasts. In the process of angiogenesis, TGF-β1 will increase the expression of VEGF for the formation of new blood vessels so that the supply of nutrients for wound healing is fulfilled. 18 In addition, TGF-β1 is able to inhibit MMP-9, resulting the inhibition of collagen degradation. 12 22 23

Applying PRP significantly decreased MMP-9 expression after 5 and 7 days. In the normal ulcer healing process, MMP-9 plays a role in the inflammatory and proliferative phases. 21 MMP-9 is a significant marker of delayed wound healing in diabetic conditions. Shin and Oh, 2010 reported that diabetic ulcers are characterized by elevated expression of MMP-9. This study was in line with a study from Shin and Oh in 2010. Shin and Oh also reported that after giving PRP, the MMP-9 expression was decreased on day 5 and day 7. 24 In diabetic conditions, MMP-9 was found to be overexpressed while TIMP-1 was under-secreted, The imbalance in the expression of MMP-9 and TIMP-1 inhibited tissue regeneration and revascularization. 21 22 In contrast, PRP containing TGF-β1 indirectly reduces the MMP-9 expression. TGF-β1 regulates TIMP-1 so that the levels of TIMP-1 increase and the overexpressed MMP-9 is inhibited by TIMP-1. 22 The clinical use of PRP in traumatic ulcers can be advantageous and disadvantageous during the ulcer healing process. The advantages of PRP are that it is it can be easily obtained from human blood and able to support hemostasis and wound healing process. 25 In contrast, the disadvantages of PRP are that it is not cost-effective and its use is partially successful. Due to these reasons, the material to be selected for treatment needs to be carefully considered to get the maximum results at an effective cost. 25 The limitation of this research is that although hyperglycemia is one of the factors that induce diabetic wounds, there are several factors in a chronic diabetic condition that can worsen a diabetic wound. Further studies are needed to analyze the effect of PRP in other marker-related chronic diabetic conditions.

## Conclusion

PRP heals traumatic ulcers in patients with diabetes mellitus by promoting the expression of TGF-β1 and suppressing that of MMP-9. This material can be a promising topical therapy for traumatic ulcers, especially with an underlying disease such as diabetes mellitus.

## References

[1] Al-Maskari A Y Al-Maskari M Y Al-Sudairy S Oral manifestations and complications of diabetes mellitus Sultan Qaboos Univ Med J 2011 11 02 179 186 21969888 PMC3121021

[2] Mauri-Obradors E Estrugo-Devesa A Jané-Salas E Viñas M López-López J Oral manifestations of diabetes mellitus. A systematic review Med Oral Patol Oral Cir Bucal 2017 22 05 e586 e594 28809366 10.4317/medoral.21655 PMC5694181

[3] Veiga N Tiago M Ana Sofia M Oral manifestations and diabetes Biomed J Sci Tech Res 2018 7 05 6168 6171

[4] Mortazavi H Safi Y Baharvand M Rahmani S Diagnostic features of common oral ulcerative lesions: an updated decision tree Int J Dent 2016 2016 7.278925E6 10.1155/2016/7278925 PMC5066016 27781066

[5] Lewis M AO Wilson N HF Oral ulceration: causes and management Pharm J 2019 302(7923):1 13

[6] Chicharro-Alcántara D Rubio-Zaragoza M Damiá-Giménez E Platelet-rich plasma: new insights for cutaneous wound healing management J Funct Biomater 2018 9 01 10 29346333 10.3390/jfb9010010 PMC5872096

[7] Tripathi R Tripathi K Management of non healing oral ulcer in diabetic patient using topical application of epidermal growth factor: a case report SAJB 2015 3 640 643

[8] Tsachiridi M Galyfos G Andreou A Autologous platelet-rich plasma for nonhealing ulcers: a comparative study Vasc Spec Int 2019 35 01 22 27 10.5758/vsi.2019.35.1.22 PMC6453601 30993104

[9] Shiga Y Orita S Kubota G Freeze-dried platelet-rich plasma accelerates bone union with adequate rigidity in posterolateral lumbar fusion surgery model in rats Sci Rep 2016 6 01 36715 27833116 10.1038/srep36715 PMC5105125

[10] Mazzucco L Balbo V Cattana E Borzini P Platelet-rich plasma and platelet gel preparation using Plateltex Vox Sang 2008 94 03 202 208 18179680 10.1111/j.1423-0410.2007.01027.x

[11] EL-Komy M H Hassan A S Abdel Raheem H M Platelet-rich plasma for resistant oral erosions of pemphigus vulgaris: a pilot study Wound Repair Regen 2015 23 06 953 955 26340377 10.1111/wrr.12363

[12] Huang Y Y Lin C W Cheng N C Effect of a novel macrophage-regulating drug on wound healing in patients with diabetic foot ulcers: a randomized clinical trial JAMA Netw Open 2021 4 09 e2122607 e2122607 34477854 10.1001/jamanetworkopen.2021.22607 PMC8417758

[13] Furman B L Streptozotocin-induced diabetic models in mice and rats Curr Protocols Pharmacol 2015 70 01 47.1, 20 10.1002/0471141755.ph0547s70 26331889

[14] Sari R P Wahjuningsi E Soeweondo I K Modulation of FGF2 after topical application of *Stichopus hermanii* gel on traumatic ulcer in Wistar rats Dent J 2014 47 03 126 129

[15] Cavalcante G M Sousa de Paula R J Souza L P Sousa F B Mota M RL Alves A PNN Experimental model of traumatic ulcer in the cheek mucosa of rats Acta Cir Bras 2011 26 03 227 234 21537526 10.1590/s0102-86502011000300012

[16] Rahman M A Sultana N Ayman U Alcoholic fixation over formalin fixation: A new, safer option for morphologic and molecular analysis of tissues Saudi J Biol Sci 2022 29 01 175 182 35002406 10.1016/j.sjbs.2021.08.075 PMC8716893

[17] Sireesh D Dhamodharan U Ezhilarasi K Vijay V Ramkumar K M Association of NF-E2 Related Factor 2 (Nrf2) and inflammatory cytokines in recent onset Type 2 Diabetes Mellitus Sci Rep 2018 8 01 5126 29572460 10.1038/s41598-018-22913-6 PMC5865120

[18] Andia I Perez-Valle A Del Amo C Maffulli N Freeze-drying of platelet-rich plasma: The quest for standardization Int J Mol Sci 2020 21 18 1 20 10.3390/ijms21186904 PMC7555364 32962283

[19] Boakye L A Ross K A Pinski J M Platelet-rich plasma increases transforming growth factor-beta1 expression at graft-host interface following autologous osteochondral transplantation in a rabbit model World J Orthop 2015 6 11 961 969 26716092 10.5312/wjo.v6.i11.961 PMC4686443

[20] Nakajima Y Kawase T Kobayashi M Okuda K Wolff L F Yoshie H Bioactivity of freeze-dried platelet-rich plasma in an adsorbed form on a biodegradable polymer material Platelets 2012 23 08 594 603 22273512 10.3109/09537104.2011.645923

[21] Huang Y Kyriakides T R The role of extracellular matrix in the pathophysiology of diabetic wounds Matrix Biol Plus 2020 6–7 100037 10.1016/j.mbplus.2020.100037 PMC7852307 33543031

[22] Li L Chen D Wang C Liu G Ran X The effect of autologous platelet-rich gel on the dynamic changes of the matrix metalloproteinase-2 and tissue inhibitor of metalloproteinase-2 expression in the diabetic chronic refractory cutaneous ulcers J Diabetes Res 2015 2015 954701 26221614 10.1155/2015/954701 PMC4499405

[23] Everts P A Knape J T Weibrich G Platelet-rich plasma and platelet gel: a review J Extra Corpor Technol 2006 38 02 174 187 16921694 PMC4680757

[24] Shin H S Oh H Y The effect of platelet-rich plasma on wounds of OLETF rats using expression on matrix metalloproteinase-2 and -9 mRNA Arch Plast Surg 2012 39 08 110 10.5999/aps.2012.39.2.106 PMC3385326 22783508

[25] Albanese A Licata M E Polizzi B Campisi G Platelet-rich plasma (PRP) in dental and oral surgery: from the wound healing to bone regeneration Immun Ageing 2013 10 01 23 23763951 10.1186/1742-4933-10-23 PMC3683340

